# Spatial distribution analysis of dentists, dental technicians, and dental therapists in Indonesia

**DOI:** 10.12688/f1000research.50869.2

**Published:** 2021-06-10

**Authors:** Nanda Rachmad Putra Gofur, Achmad Zam Zam Aghasy, Aisyah Rachmadani Putri Gofur

**Affiliations:** 1 Department of Health, Faculty of Vocational Studies, Universitas Airlangga, Surabaya, Indonesia; 2 Indonesian Health Collaboration and Innovation Institute, Surabaya, Indonesia; 3 Faculty of Dental Medicine, Universitas Airlangga, Surabaya, Indonesia

**Keywords:** Healthcare, Public health, Health service, Health worker density

## Abstract

**Background: **Access to health services is needed around the world, from healthcare providers to doctors. One of the needs in public health is a system that is accessible for everyone, but, unequal distribution of healthcare provider and health workers, especially in dentistry fields is still a main problem in several countries, including Indonesia. The aim of this study is to analyze the spatial distribution of dentists, dental technicians, and dental therapists.

**Methods: **This spatial analysis study was conducted after obtaining secondary data in Indonesia. All data were collected between September 1 ^st^, 2020 and October 1 ^st^, 2020 from open access sources of de-identified data. The data of dentists per area, dental technicians per area, and dental therapists per area were calculated for analysis. A spatial distribution map was prepared using the Quantum Geographic Information System (QGIS Desktop, version 3.10.6).

**Results: **The results of this study found a ratio of dentists to members of the population in Indonesia of 1:17,105. The average number of dental technicians that work in the public health centers in each province (dental technicians per area) in Indonesia was calculated to be 0.13. The average number of dental therapists that work in the public health centers in each province (dental therapists per area) in Indonesia was calculated to be 0.40. This spatial autocorrelation illustrates that there is a relationship between values of dentists per area and dental therapists per area between provinces in Indonesia, and shows geographic clustering relationships or patterns that are grouped and have similar characteristics in adjacent locations. This spatial autocorrelation did not occur in the value of dental technicians.

**Conclusions: **From this study we can conclude that there is an unequal distribution of dental personnel in Indonesia.

## Introduction

Indonesia is a country in Southeast Asia located between Indian and Pacific oceans. It has more than 17,000 islands, but the biggest islands are Sumatra, Java, Borneo or Kalimantan, Sulawesi and Papua. Indonesia is the 14 ^th^ largest country in the world with a land area of 1,904,569 square kilometers or 735,358 square miles and is the world’s largest island country. Indonesia is bordered by several country such as Malaysia, East Timor, Papua New Guinea, Singapore, Vietnam, Philippines, Palau and Australia ^1^.

Based on the Global Burden of Disease (GBD) study in 2016 ^2^, dental and oral health problems, especially dental caries, affects nearly half of the world's population (3.58 billion people). Gum disease (periodontal) is the 11th most common oral health disease in the world. Meanwhile, in Asia Pacific, oral cancer is the third most common type of cancer. The results of the Basic Health Research (Riskesdas) in 2018 stated that 57.6% of the Indonesian population had dental and oral problems during the last 12 months, but only 10.2% received treatment by dental medical personnel (dental nurses, dentists or specialist dentists), while the rest received no treatment ^2, 3^. The largest proportion of dental problems in Indonesia are dental caries, necrosis tooth, and toothache complaints (45.3%). Meanwhile, the majority of oral health problems experienced by Indonesians are swollen gums (14%) ^3, 4^.

Effective Medical Demand (EMD) is defined as the percentage of the population who had oral health problems in the last 12 months multiplied by the percentage of the population who received dental care or treatment from dental medical personnel (specialist, dentists, dental nurses/dental therapists) and dental technicians ^5^. The EMD in Indonesia is only 8.1%, it means that only few of the population receive dental treatments when they have dental problems. The overall ability to get services from dental medical personnel (EMD) in Indonesia is only 8.1% ^5, 6^. Three provinces of Indonesia in 2013, as South Sulawesi, South Kalimantan and Central Sulawesi had quite high levels of oral and dental problems (>35%), with EMD of 10.3%, 8.0%, and 6.4%, respectively ^7^. Based on this finding, a clear picture of the gap between dental problems and the EMD that occur in society can be seen.

A public health system is needed, whereby everyone can access public health services, including dental services, at an affordable rate including affordability of health services. Private health-care providers might help affordability of health services beside government health services. The main focus which needs attention is on curative care in single-practitioner and group practices in dentistry fields. But, unfortunately, these private health services are driven by market demands in society and are business-oriented services. The Geographic Information System (GIS) is a method commonly used in health research. This method could analyze various healthcare variables related to other fields such as physical, social and cultural environments ^8, 9^. Access to health services is very important and needed around the world. The correlation between the service provider (the health practitioner) and the consumer (the patient) is still a main problem in many other countries including Indonesia ^10, 11^.

As of 2012, from 8,975 public health centers, there are 5,439 public health centers with dentists and 3,536 public health center without dentists. Nationally, 47.4% of public health centers had one dentist and 13.2% of public health centers had more than two dentists ^12, 13^. Public health centers that have the highest efforts in dental and oral health service is in the Bali province, which is 100% and the lowest efforts in dental and oral health service in Indonesia is in Papua Province, which is 24%; the national rate for this efforts of dental and oral health services is 84%. This means, Bali is the province that is best at providing dental services and appropriately considers dentistry fields, meanwhile Papua is the worst ^14, 15^.

The residents ease to reach services and facilities based on the distance and travel time to a resource is called geographic accessibility. Optimal delivery of dental health services should take into consideration availability and accessibility, which together are referred to as spatial accessibility ^16– 18^. The aim of this study was to analyze the spatial distribution of dentists, dental technicians, and dental therapists in Indonesia.

## Methods

This spatial analysis study was conducted after obtaining secondary data in Indonesia. All data were collected between 1 ^st^ September 2020 and 1 ^st^ October 2020 from open access sources of de-identified data; therefore, no ethical approval was required. We sought to identify the average number of dentists, dental technicians and dental therapists working in public sectors and the main building of public health centers in each province.

This study retrieved data of the number of dentists in each province in 2018 from the national Indonesian Health Facility Research report (Riset Fasilitas Kesehatan), accessed via the Ministry of Health, Indonesia.

The geographic administrative area data of Indonesia were retrieved from the Indonesia Geospatial Portal, which is publicly available. The provincial maps of the Indonesian database were processed with the Geospatial Portal for further geographic information system (GIS) based analysis to identify spatial distribution of dentists, dental technicians, and dental therapists per area by obtaining the province level polygon map that contains information regarding latitudes and longitudes of each province. We used the map of all 34 provinces of Indonesia for the analysis. Data on the distribution of dentists, dental technicians, and dental therapists was collected from the Ministry of Health and Geospatial Portal.

We created a map using the Quantum Geographic Information System (QGIS Desktop, version 3.10.6). Global and local Moran’s indices were calculated to determine the autocorrelation value and local indicator of spatial autocorrelation (LISA). LISA was analyzed using GeoDa software, version 1.10.0.8. The level of significance was set at p-value of <0.05, Z _α/2_ at 1.96, and randomization run to 999 permutations. We used automatic Euclidean weight distance, which matched with the assumption that each province has at least four neighboring provinces. Interpretation of the LISA significance map includes the following categories:

- “high–high” indicates a clustering of high value rates (positive spatial autocorrelation)
- “low–high” indicates that the low value rates are adjacent to high value rates (negative spatial autocorrelation)
- “low–low” indicates clustering of low value rates (positive spatial autocorrelation)
- “high–low” indicates that high value rates are adjacent to low value rates (negative spatial autocorrelation)
- “not significant” indicates that there is no spatial autocorrelation.

The outcome of Moran’s I identifies the intensity of spatial autocorrelation along with the result of statistically significant test, that is, the p value. The following mathematical representation exhibits the computation of Moran’s I:


$$\begin{array}{c} I\;\;=\;\;\frac{N}{S_{0}}\frac{\sum_{i=1}^{N}\sum_{j=1}^{N}W_{ij}(x_{i}\;–\;\bar{x})(x_{j}\;–\;\bar{x})}{{\sum_{i=1}^{N}\left(x_{i}\;–\;\bar{x})(x_{i}\;–\;\bar{x}\right)}^{2}}\\ S_{0}\;\;=\;\sum_{i=1}^{N}\sum_{j=1}^{N}W_{ij} \end{array}$$

Where W _ij_ is the spatial weight between the parameters in provinces i and j. The parameters are the number of dentists per area, dental technicians per area, and dental therapists per area. N is the total number of spatial units; S0 is the aggregate of all spatial weights; x _i_ and x _j_ are the amount of each dental health personnel in provinces i and j, respectively.

## Results and discussion

The total number of public health centers in Indonesia were counted at 9,831. The total number of dentists, dental technicians, and dental therapists that work in public sectors of public health center main buildings in each province was 15,833, 1,214, and 3,834, respectively ( Table 1).

**Table 1.** Distribution of dental personnel in each province in Indonesia.

Province	Total Public health center (n)	Dentist		Dental Technician		Dental Therapist	
		Total	Average of each public health center work area	Total	Average of each public health center work area	Total	Average of each public health center work area
Aceh	347	414	1,19	92	0,27	145	0,42
North Sumatera	571	900	1,58	64	0,11	60	0,11
West Sumatera	271	544	2,01	38	0,14	139	0,51
Riau	216	555	2,57	25	0,12	38	0,18
Jambi	193	287	1,49	29	0,15	100	0,52
South Sumatera	328	362	1,10	36	0,11	259	0,79
Bengkulu	179	129	0,72	15	0,08	7	0,04
Lampung	299	238	0,80	27	0,09	87	0,29
Bangka Belitung	63	113	1,79	24	0,38	50	0,79
Kepulauan Riau	80	138	1,73	4	0,05	31	0,39
DKI Jakarta	313	1416	4,52	15	0,05	115	0,37
West Java	1069	1915	1,79	168	0,16	451	0,42
Central Java	876	1517	1,73	89	0,10	428	0,49
DI Yogyakarta	121	390	3,22	28	0,23	132	1,09
East Java	964	2318	2,40	112	0,12	324	0,34
Banten	233	535	2,30	23	0,10	24	0,10
Bali	120	640	5,33	13	0,11	206	1,72
West Nusa Tenggara	161	198	1,23	49	0,30	103	0,64
East Nusa Tenggara	374	210	0,56	27	0,07	252	0,67
West Kalimantan	241	197	0,82	39	0,16	240	1,00
Central Kalimantan	197	147	0,75	38	0,19	81	0,41
South Kalimantan	232	264	1,14	49	0,21	118	0,51
East Kalimantan	178	341	1,92	8	0,04	51	0,29
North Kalimantan	55	85	1,55	9	0,16	11	0,20
North Sulawesi	193	128	0,66	20	0,10	82	0,42
Central Sulawesi	196	171	0,87	31	0,16	21	0,11
South Sulawesi	452	844	1,87	57	0,13	203	0,45
Southeast Sulawesi	281	219	0,78	41	0,15	31	0,11
Gorontalo	93	89	0,96	5	0,05	4	0,04
West Sulawesi	94	108	1,15	12	0,13	14	0,15
Maluku	199	90	0,45	9	0,05	10	0,05
North Maluku	129	102	0,79	2	0,02	3	0,02
West Papua	157	54	0,34	5	0,03	12	0,08
Papua	356	175	0,49	11	0,03	2	0,01
Indonesia	9831	15833	1,61	1214	0,13	3834	0,40

**Figure 1.**
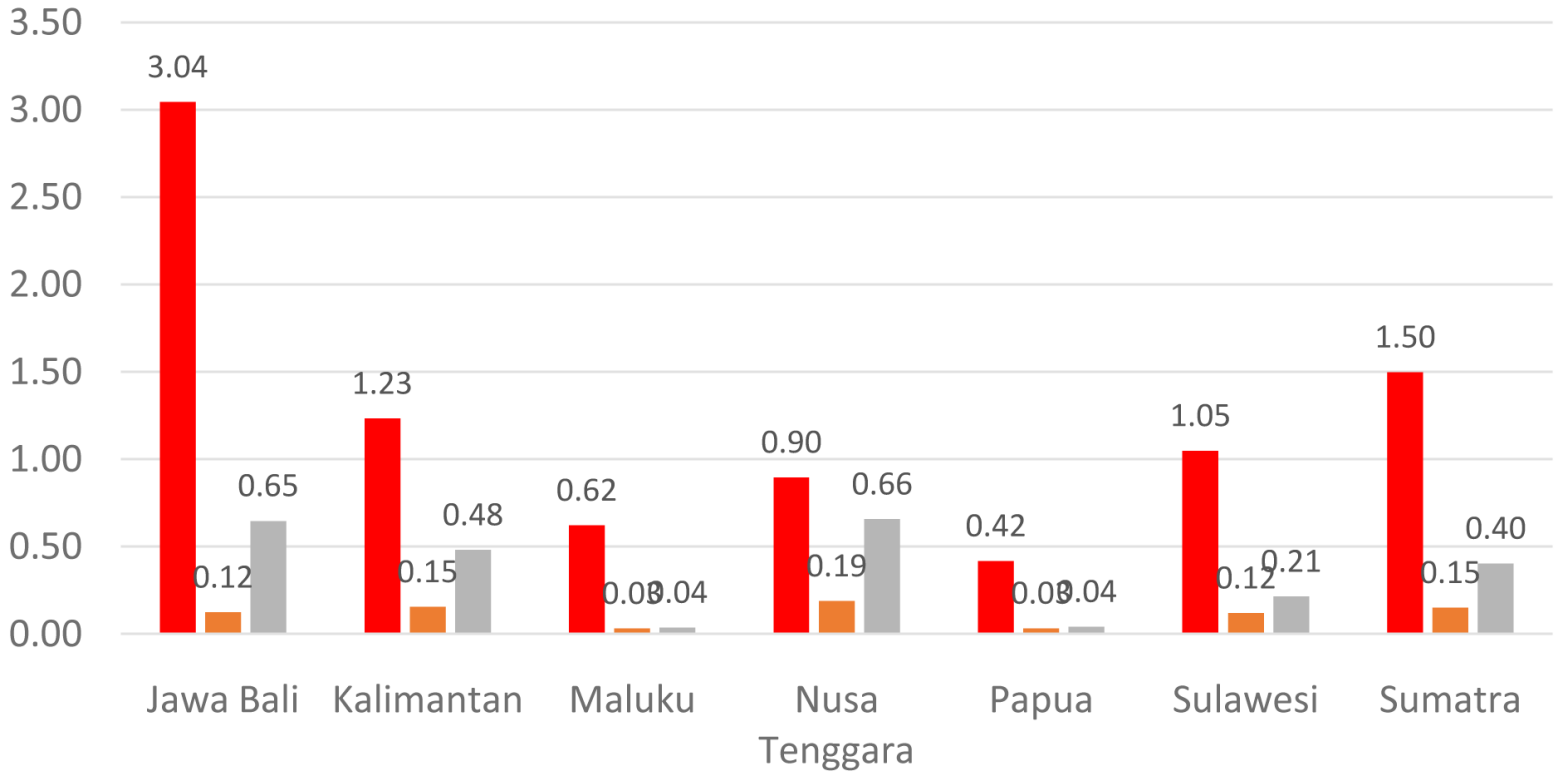
Distribution of dentists (red), dental therapists (orange) and dental technicians (grey) in Indonesia (x axis is island name in Indonesia; y axis is the average number of dental health personnel per public health center).

**Figure 2.**
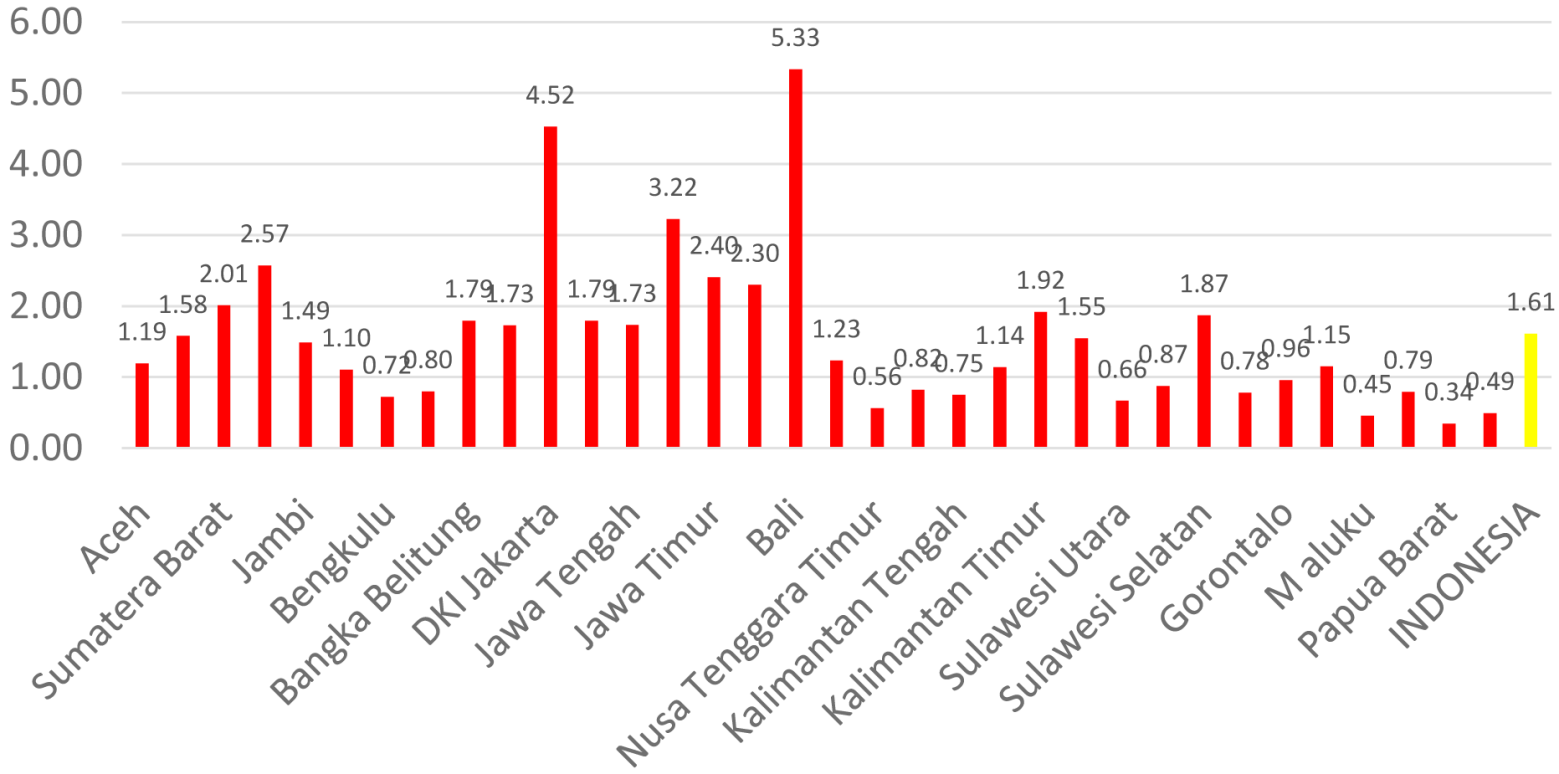
Average number of dentists at every Province in each public health center.

**Figure 3.**
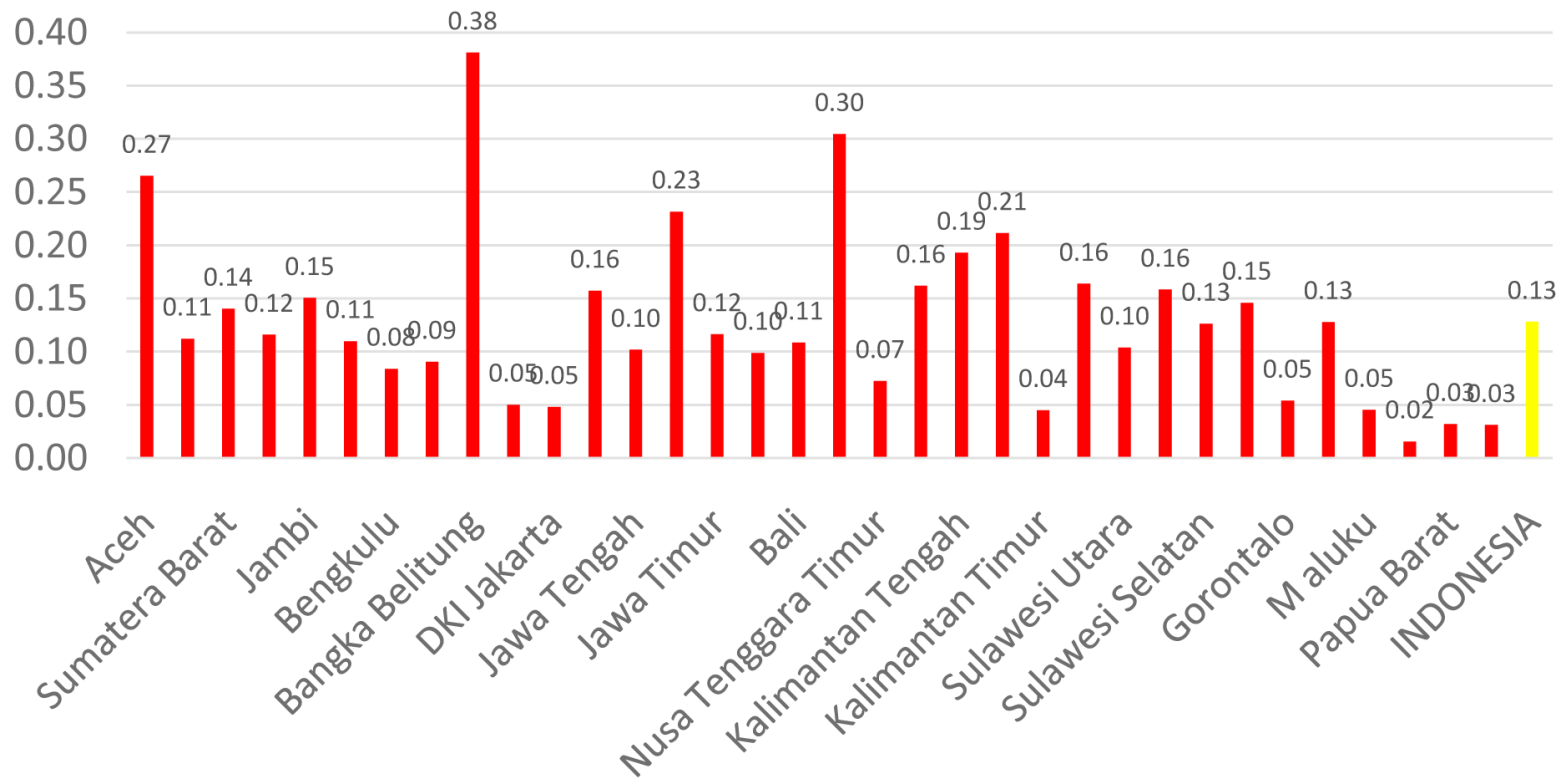
Average number of dental therapists at every Province in each public health center.

**Figure 4.**
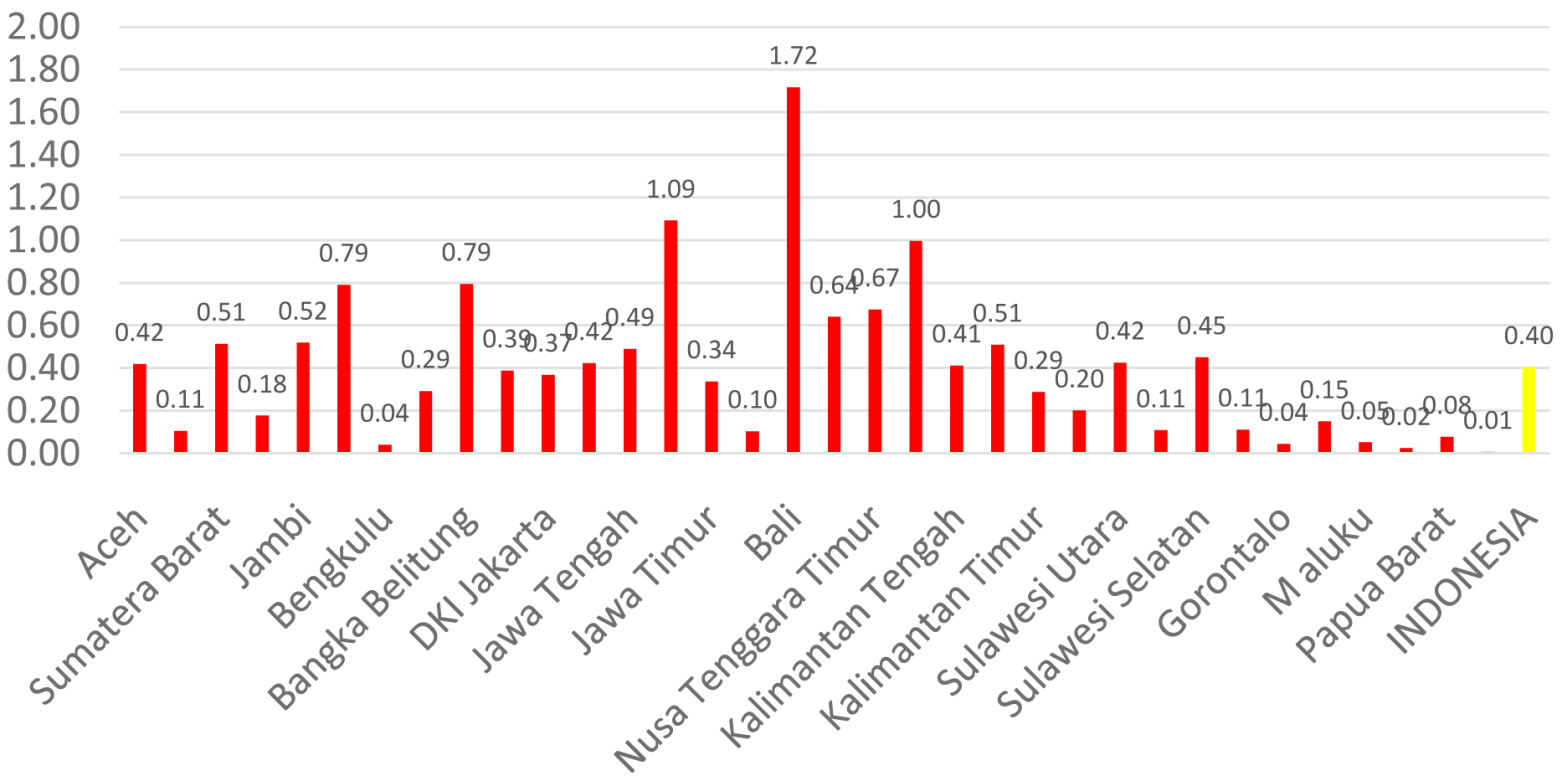
Average number of dental technicians at every Province in each public health center.

Figure 1 shows the distribution of dental health personnel in several major islands in Indonesia. The distribution is still unequal due to the big difference in the amount of dental personnel in each island. The average number of dentists that work in public sectors and public health centers in each province (dentist per area) in Indonesia was calculated at 1.61 ( Figure 2). The ratio of dentist to members of the population in Indonesia was calculated at 1:17,105. The average number of dental therapists that work in main buildings of public health centers in each province (dental therapist per area) in Indonesia was calculated at 0.40 ( Figure 3). The average number of dental technicians that work in main buildings of public health centers in each province, (dental technicians per area) in Indonesia were calculated at 0.13 ( Figure 4).

There are 13 provinces that have a value of dentists per area below 1, which indicates that there are areas that do not have dentists at all in public health centers. All provinces in Indonesia have a value of dental technicians per area below 1, which shows that all regions of Indonesia do not have sufficient numbers of dental technicians for each public health center area. There are 31 provinces that have a value of dental therapists per area below 1, which indicates that there are areas that do not have dental therapists at all in a health center area ( Figure 1– Figure 4).

**Figure 5.**
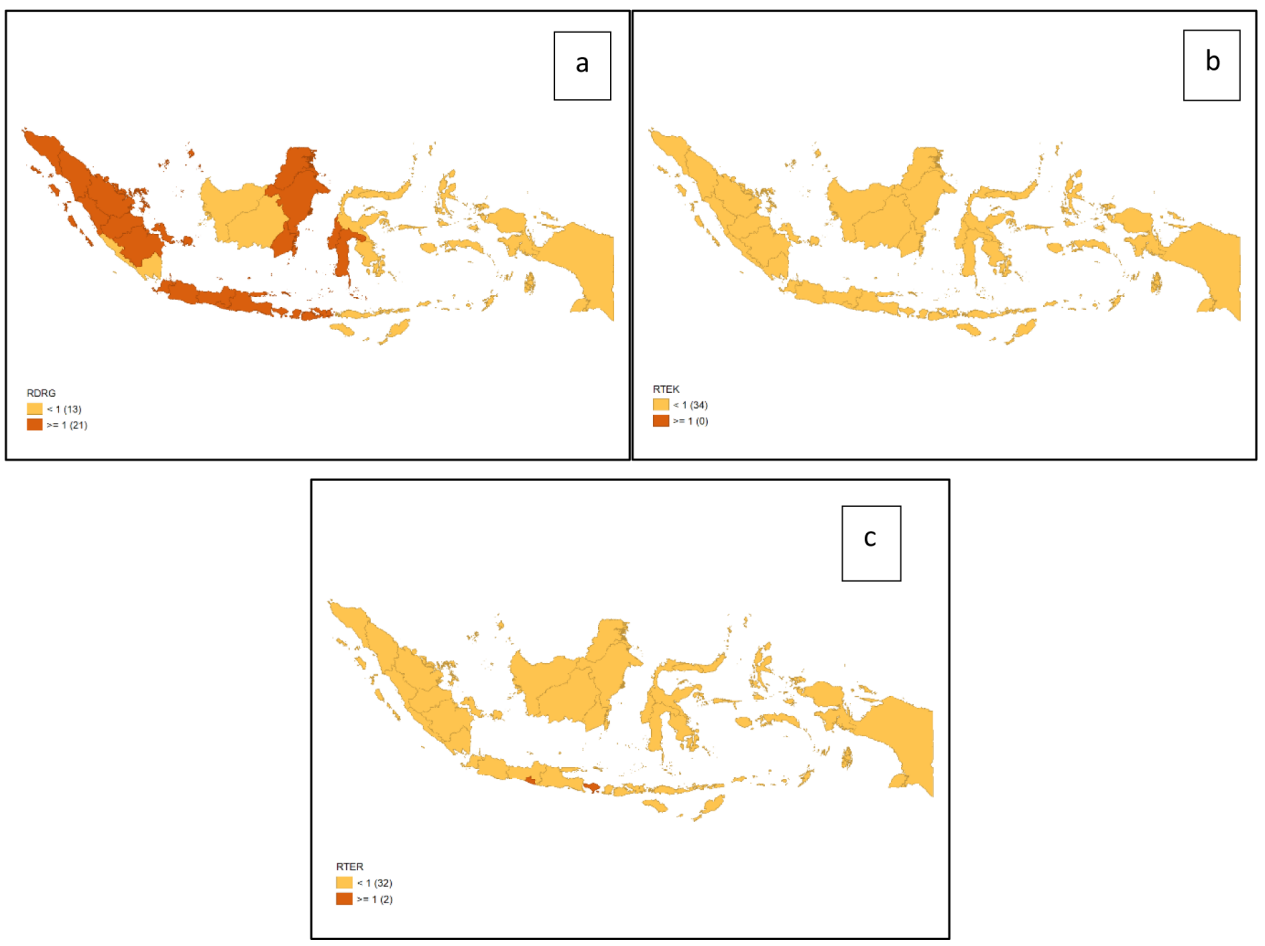
Visualization maps of dentists per area ( **a**), dental therapists per area ( **b**), and dental technicians per area ( **c**). Orange = <1; red = ≥1.

The highest number of dentists per distribution area is in the province of Bali, Java-Bali, and the lowest is in the province of West Papua, Papua. The highest number of dental technicians per distribution area is in the province of Bangka Belitung, Sumatra, and the lowest is in the province of North Maluku, Maluku. The highest number of dental therapists per distribution area is in the province of Bali, Java-Bali, and the lowest is in the province of Papua. Visualization maps of dentists, dental technicians, and dental therapist per area were developed and are presented in Figure 5.

Indonesia had both a shortage of and an unequal distribution of dental health personnel in public health center areas. The distribution of dentists is similar to that in other studies, which have indicated maldistribution of both general practitioners (GPs) and specialists ^19^. Maldistribution indicates inequality associated with area. Dentist and dental nurses are two health workers who play an important role in providing dental health services. The distribution of the two health workers was the highest in the Java-Bali region (60.1%), and the lowest in Papua region (10.3%). However, there are a high number of dentists and dental therapists in the Maluku Islands region (57.9%) ^11, 12^. Based on this, it appears that there is a gap that occurs between regions related to the availability of dentists and dental therapists on duty at the health centers. These results are also supported by a previous study that found an unequal distribution ^19– 21^.

The poor distribution of health workers is not only present in dental health service personnel, but in other types of health workers too. Although there is a health worker placement policy in Indonesia which uses a temporary employee system for medical personnel (doctors and dentists), the distribution is not equal especially in remote areas. From the results of the analysis conducted by Ihsan Husain, *et al.* (2006), on average there are more dentists in Java-Bali compared to the other regions ^10, 15^.

Unequal distribution of dentists can be seen from comparing the actual number versus ideal number of dentists with the total population. The ideal ratio is one dentist for 9,090 residents. In the year of 2010, based on dentist registration data from the Indonesian Medical Council (KKI), there were 22,237 registered dentists consisting of 20,665 general dentists and 1,582 specialist dentists ^7^. However, not all dentists that were registered at the Indonesia database work in a public health center. That means only 60.6% of dentists work for an Indonesian public health center, and the rest works in private sector. The amount of Dentists who work in the private sector is 49.4% and they might not be distributed equally around Indonesia ^9^.

Many factors cause this inequality, including the placement policy of health workers in each region (province and district / city), the quality and number of health service facilities, shifting disease patterns, especially in urban regions, high disparity in health status community between regions, and characteristics of the geographic area. From the results of a study conducted by Bappenas, there is a gap between the number of workers and public health centers who need health workers, in that the number of workers is higher than public health structure itself. Furthermore, to ensure health workers are evenly distributed, it is necessary to develop an even infrastructure ^10– 12^.

Health worker placement policy is largely determined collectively by health offices and regional civil service agencies. According to a result from a study in two districts in the province of Gorontalo, the unequal distribution of healthcare workers occurs due to lack of healthcare facilities. Increasing the number of health workers every year was not followed by an equal distribution to health facilities. The study also found that there were three factors that affected the policies and development of health facilities, namely disparities of health status, migration of population, and mutual geographic characteristics ^10, 15^.

**Figure 6.**
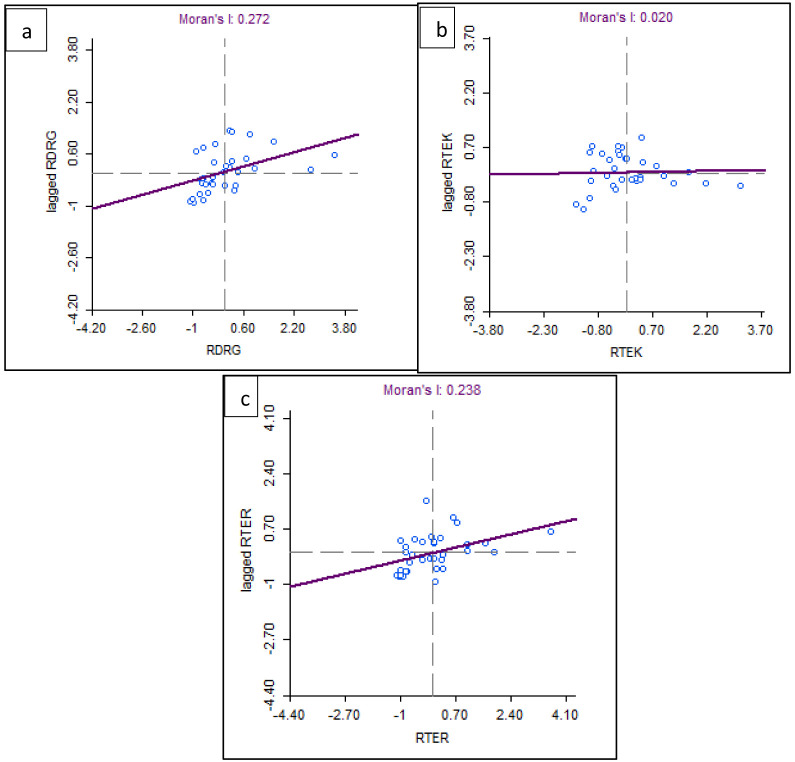
Spatial autocorrelation with Moran’s index of dentists per area ( **a**), dental therapists per area ( **b**), and dental technicians per area was developed ( **c**). The X axis is referring to the number of observations and is also known as the response axis. The Y axis is referring to the average or spatial lag of the corresponding observation of dental health personnel per area of the X axis.

Based on the results of the spatial autocorrelation test with Moran's I, there was a significant spatial autocorrelation of dentists per area (I: 0.272, z-value: 3.20) and dental therapists per area (I: 0.238, z-value: 2.85) in Indonesia. Meanwhile, the value of dental technicians per area (I: 0.002, z-value: 0.47) did not show significant spatial autocorrelation ( Figure 6).

According to the World Health Organization (WHO), the ideal ratio of dentists for an area is 1:8,000 people, while Indonesia currently stands at 1:17,105. Dentists per area in Indonesia have spatial autocorrelation at the provincial level and are concentrated only in the Java-Bali region, with very few in the Papua region. For example, Saudi Arabia is one of the countries that has improved the ratio of dentists to population. In 1987 the ratio of dentists to population was 1:8,906, but in 2016 this number increased to 1:1,880. The ratio of dentists to population in Saudi Arabia recently was 5.3 per 10,000 people. This number is higher than all the developing countries in Asia-Pacific region. Also, in this region, China reported the lowest ratio of dentists to population, with 0.12 per 10,000 people. And on the other side, Japan has the highest ratio of dentists to population ratio in Asia-Pacific region with 7.7 per 10,000 people ^18, 22, 23^.

Other studies reported that the OECD member countries, excluding Scandinavian countries and Greece, have varied dentists-to-population ratios ranging from 5 to 8 with the average of 6.1 per 10,000 people. In addition, most of the European countries have dentists-to-population ratios ranging from 5.07 to 7.3 per 10,000 people. Among Middle Eastern countries, Bahrain has the lowest dentists-to-population ratio of 1.5 per 10,000 people, and Qatar has the highest dentists-to-population ratio of 5.8 per 10,000 people ^19, 22, 24, 25^.

Based on the distribution pattern that we found, we assumed that the distribution of dentists in Indonesia occurred due to the large population, the number of dental institutions, as well as the equitable distribution of development in each region. Java island is one of the areas with an equal distribution of dentists in Indonesia, though it is also the most populated island in Indonesia ^20, 21^. In line with the large population growth, Java Island is an area that has 18 dental institutions out of a total of 31 dental institutions throughout Indonesia. Issues of development and availability of public facilities and infrastructure are the main reasons why dentists prefer to practice in the Java area. Dental therapists per area also have spatial autocorrelation at the provincial level, where the Nusa Tenggara region has the highest distribution and the Papua region has the least ^26, 27^.

**Figure 7.**
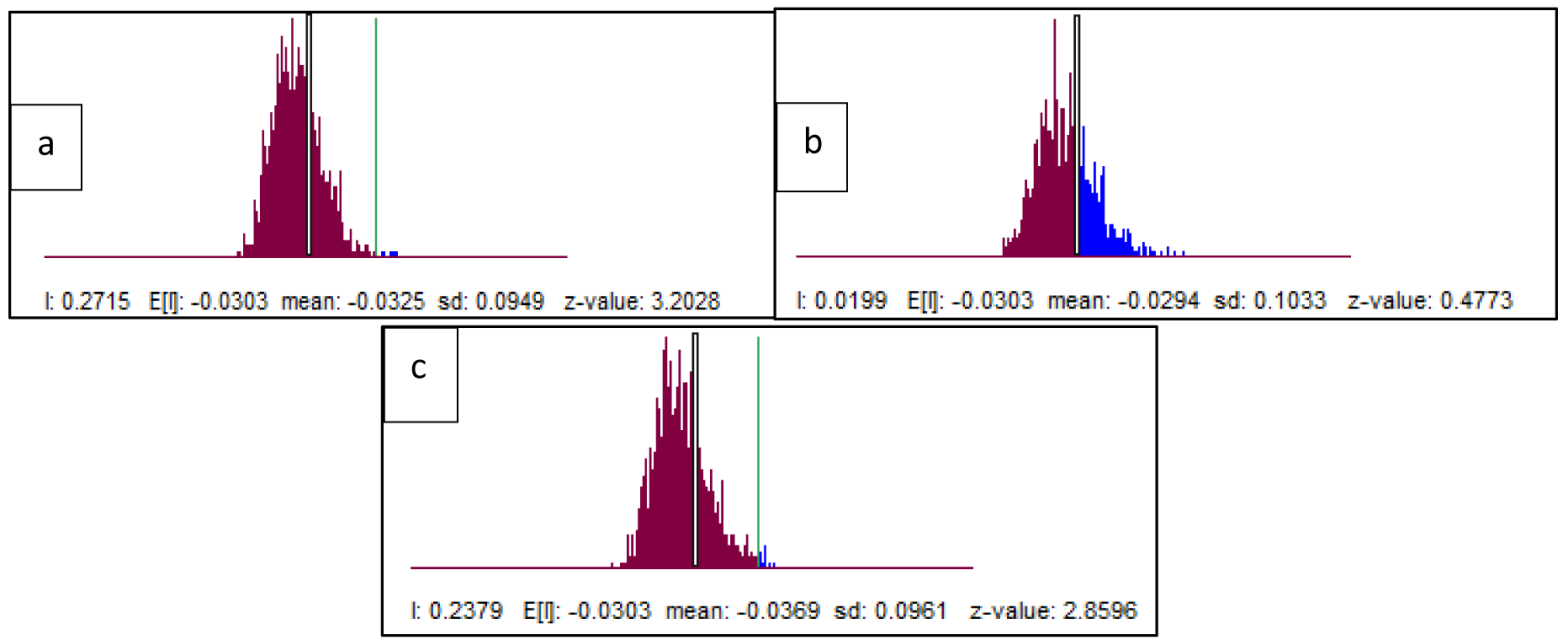
Spatial autocorrelation illustration of dentists per area ( **a**), dental therapists per area ( **b**), and dental technicians per area ( **c**). The plots show the spatial autocorrelation between the number of dentists and dental therapists per area in every province in Indonesia. But the dental technician is not showing the spatial autocorrelation.

This spatial autocorrelation illustrates that there is a correlation between the number of dentists per area and dental therapists per area between provinces in Indonesia and shows geographic clustering relationships or patterns that are grouped and have similar characteristics in adjacent locations. This spatial autocorrelation did not occur in the value of dental technicians. The results of Moran's I test show that there is an autocorrelation or spatial relationship of the number of dentists per area and dental therapists per area in Indonesia. Furthermore, Moran's scatterplot also illustrates the pattern of relationships between the existing provinces ( Figure 7).

Based on the distribution patterns, similar to what happened in the maldistribution of dentists, the maldistribution that occurs in dental therapists may also be due to the population number, the number of dental institutions, and the equal distribution of welfare in each region according to this result. In addition, we assumed that the geographical grouping of dental therapists in the Nusa Tenggara region was due to the process of substituting the role of dentists by dental therapists where the distribution of dentists in the area was low. Meanwhile, a very minimal distribution was seen in the value of dental technicians per area. In this study, it was found that all regions of Indonesia did not have a sufficient number of dental technicians for each public health center, with a mean value of less than 1 per public health center. The factor that most influences this is the very limited number of schools for dental technicians; only 10 institutions throughout Indonesia. Several conditions above then describe the minimum distribution of dental personnel in Indonesia ^28^.

A sufficient number of dental health personnel, such as dentists, dental technicians, and dental therapists in a Province is very important in efforts to ensure health service. The Indonesian government has made regulations regarding the minimum number of dentists in public health centers (minimum 1), but there is not a distribution policy for dental technicians and dental therapists. Indonesia has also implemented various policies, such as increased numbers of dental students to be trained in faculties of dentistry nationwide and pre contract for dentists to work in rural areas after graduation through the Nusantara Sehat and PTT Daerah programs, to solve the shortage of dentists. However, the inequality of dental personnel distribution is still being found ^29^.

**Figure 8.**
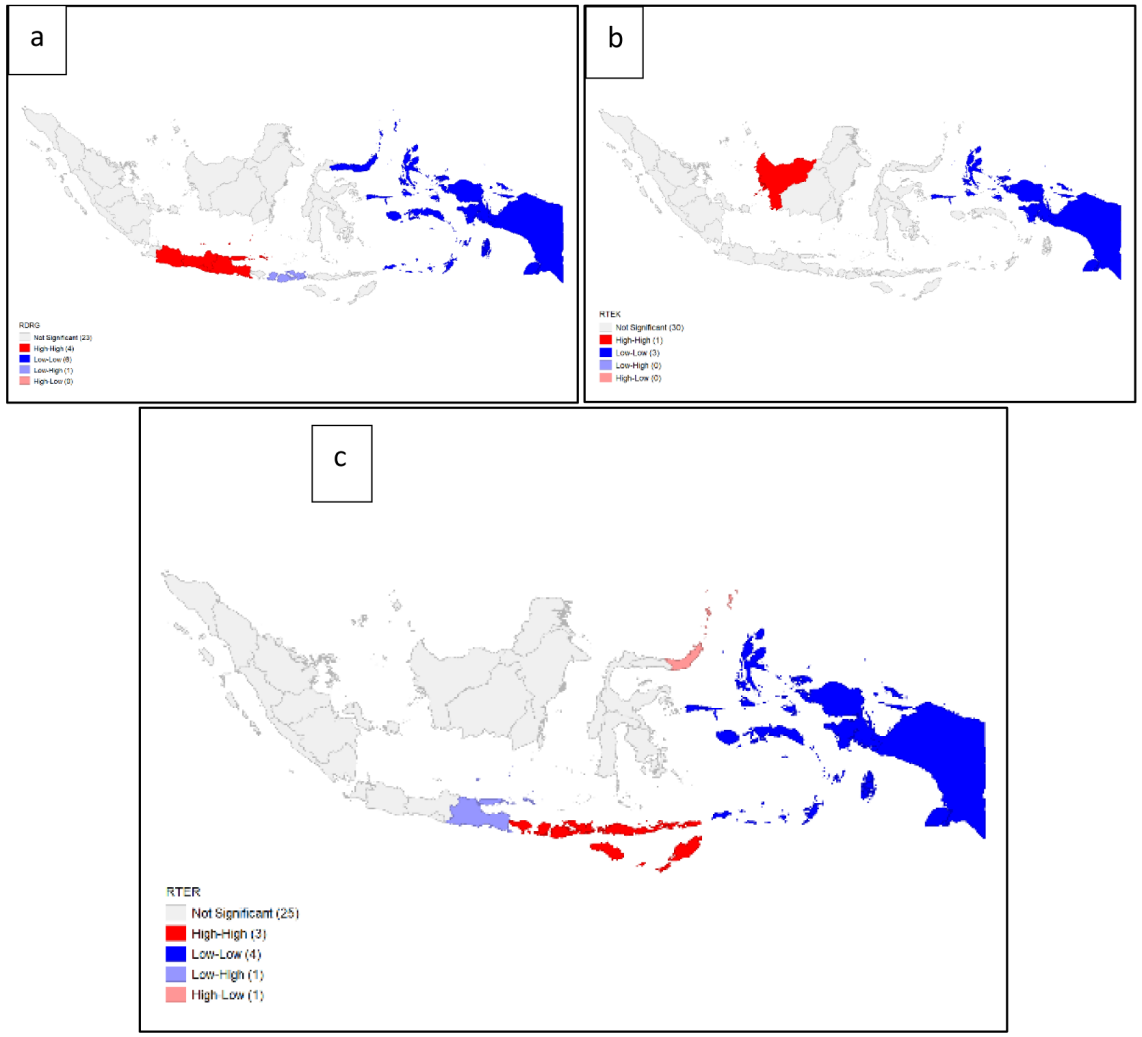
Autocorrelation local indicator of spatial autocorrelation (LISA) map of dentists per area ( **a**), dental therapists per area ( **b**), and dental technicians per area ( **c**). Grey = not significant, red = high-high, blue = low-low, purple = low-high, peach = high-low.

We determined the significance of local spatial autocorrelation through LISA. From this test, the significance of the relationship in each province was obtained. The pattern of Moran's local significance levels is presented in Figure 8. Interpretation of the LISA significance map includes the following categories:

“High-high” indicates a clustering of high-value rates (positive spatial autocorrelation)

“Low-high” indicates that the low-value rates are adjacent to high-value rates (negative spatial autocorrelation)

“Low – low” indicates clustering of low-value rates (positive spatial autocorrelation)

“High – low” indicates that high-value rates are adjacent to low-value rates (negative spatial autocorrelation)

“Not significant” indicates that there is no spatial autocorrelation.

Spatial analysis was used to identify dental health personnel shortages and geographical distribution. The traditional methods use administrative boundaries such as counties as the basic spatial units as well as dental health personnel to identify shortages based on those numbers ^30– 32^. Such approaches have been criticized for their inability to account for either the spatial variations of population demand and dental health personnel supply within those boundaries or for population–dental health personnel interactions across them. Spatial analysis can assist current and future dental health personnel, dental school administrators, and policymakers in making informed decisions to determine suitable practice locations, dental school admissions criteria, and target areas for public health initiatives ^33, 34^.

The problem of unequal distribution of dental health personnel should use spatial analysis to understand the issues. This study included number of dental health personnel in each public health center for the accuracy of this research. This research can be accurate because the characteristics of public health centers in each region are similar ^35– 37^. Future studies are recommended to use data of dental health personnel both in the public sector and in the private sector.

## Conclusion

The number of dentists in Indonesia has increased due to the increase of dental students who have been trained in faculties of dentistry which are also done the pre-contracts for dentists to work in rural areas after graduation through the Nusantara Sehat and PTT Daerah programs, to solve the shortage of dentists. However, the inequality of dental personnel distribution is still found, there is no distribution policy for dental technicians and dental therapists, and the dentist-to-population ratios in Indonesia have not improved. Spatial analysis might help identify dental health personnel shortage and geographical distribution.

## Data availability

### Source data

The distribution data of the number of dentists in each province is available at https://www.litbang.kemkes.go.id/ ( Ministry of Health) and geographic area data is available from https://tanahair.indonesia.go.id/portal-web ( Geospatial Portal).
