# Supplementary material for: Spatial distribution analysis of dentists, dental technicians, and dental therapists in Indonesia
Source: F1000Res. 2021 Jun 10;10:220. Originally published 2021 Mar 18. [Version 2] doi: 10.12688/f1000research.50869.2 (PMC8150122; doi:10.12688/f1000research.50869.2)
Supplement: Peer review history

# Peer review: Reviewer response for version 2

**DOI:** 10.5256/f1000research.57637.r87316

**Published:** 2021-06-11

**Authors:** Alessandro Leite Cavalcanti

**Affiliations:** 1 Department of Dentistry, UEPB - Universidade Estadual da Paraíba, Campina Grande, Brazil

No further comments.

Is the work clearly and accurately presented and does it cite the current literature?

Yes

If applicable, is the statistical analysis and its interpretation appropriate?

Yes

Are all the source data underlying the results available to ensure full reproducibility?

Yes

Is the study design appropriate and is the work technically sound?

Yes

Are the conclusions drawn adequately supported by the results?

Yes

Are sufficient details of methods and analysis provided to allow replication by others?

Yes

Reviewer Expertise:

Public Health and Epidemiology

I confirm that I have read this submission and believe that I have an appropriate level of expertise to confirm that it is of an acceptable scientific standard.

# Peer review: Reviewer response for version 1

**DOI:** 10.5256/f1000research.53958.r81866

**Published:** 2021-05-25

**Authors:** Alessandro Leite Cavalcanti

**Affiliations:** 1 Department of Dentistry, UEPB - Universidade Estadual da Paraíba, Campina Grande, Brazil

I congratulate the authors for the excellent quality of the manuscript. The study is relevant and describes the profile of dental professionals in Indonesia. Some minor corrections are suggested:

1. Page 1: ABSTRACT: It is not necessary to repeat the name of the country in the results, since it is already in the objective.
2. Page 3, left column, 2nd paragraph, line 11: I suggest excluding "while the rest received no treatment".
3. Page 3, right column, 1st paragraph, sentence 1: New wording "As of 2012, from 8,975 public health centers, there are 5,439 public health centers with dentists (60.6%)." You must delete "and 3,536 public health center without dentists".
4. Page 6, left column, 2nd paragraph: I suggest that the authors inform how many dental schools there are in the country and how many dentists are trained each year. The same applies to dental therapists and technicians.
5. Page 10: I recommend that the authors describe the limitations of the study.

Is the work clearly and accurately presented and does it cite the current literature?

Yes

If applicable, is the statistical analysis and its interpretation appropriate?

Yes

Are all the source data underlying the results available to ensure full reproducibility?

Yes

Is the study design appropriate and is the work technically sound?

Yes

Are the conclusions drawn adequately supported by the results?

Yes

Are sufficient details of methods and analysis provided to allow replication by others?

Yes

Reviewer Expertise:

Public Health and Epidemiology

I confirm that I have read this submission and believe that I have an appropriate level of expertise to confirm that it is of an acceptable scientific standard.

# Peer review: Reviewer response for version 1

**DOI:** 10.5256/f1000research.53958.r85400

**Published:** 2021-05-17

**Authors:** Reny Purnama Hadi

**Affiliations:** 1 Master Program in Biomedical Science, Faculty of Medicine, Brawijaya University, Malang, Indonesia

First of all the article is unique, never discussed before and adequate. Moreover, there are some additional suggestions: 

1. The abstract background needs to focus on spatial distribution; 3 professions.
2. The method is adequate and clear.
3. The first sentence of the conclusion should be answering on the background.
4. The discussion is clear and adequate.

After all, the overall is very good.

Is the work clearly and accurately presented and does it cite the current literature?

Yes

If applicable, is the statistical analysis and its interpretation appropriate?

Yes

Are all the source data underlying the results available to ensure full reproducibility?

Yes

Is the study design appropriate and is the work technically sound?

Yes

Are the conclusions drawn adequately supported by the results?

Yes

Are sufficient details of methods and analysis provided to allow replication by others?

Yes

Reviewer Expertise:

Biomedical, Public Health of Medicine

I confirm that I have read this submission and believe that I have an appropriate level of expertise to confirm that it is of an acceptable scientific standard.
